# Understanding peer mentorship in supporting self‐management of hip and knee osteoarthritis: A qualitative study of mentees' perspectives

**DOI:** 10.1002/msc.1580

**Published:** 2021-07-27

**Authors:** Elizabeth C. Lavender, Anna M. Anderson, Esther Dusabe‐Richards, Deborah Antcliff, Sarah R. Kingsbury, Philip G. Conaghan, Gretl A. McHugh

**Affiliations:** ^1^ School of Healthcare University of Leeds Leeds UK; ^2^ Leeds Institute of Rheumatic & Musculoskeletal Medicine University of Leeds and NIHR Leeds Biomedical Research Centre Leeds UK; ^3^ NIHR Leeds Biomedical Research Centre Leeds UK; ^4^ Physiotherapy Department Bury & Rochdale Care Organisation Northern Care Alliance NHS Group Bury UK

**Keywords:** intervention, mentee, osteoarthritis, qualitative study, peer mentorship, self‐management

## Abstract

**Background:**

Hip and knee osteoarthritis (OA) are common musculoskeletal conditions. Treatment is usually conservative, making self‐management a priority. We developed and trialled an OA peer mentorship intervention to support self‐management in older people. Our objectives were to gain understanding of the perceived challenges of living with OA and explore how a peer mentorship intervention can support tackling these challenges; and to explore mentees' experiences of receiving the intervention to understand how this affected their OA self‐management.

**Methods:**

Qualitative semi‐structured interviews focussing on acceptability and feasibility of being in the study were conducted with mentees. Transcribed interviews were double coded and subject to framework analysis. To address the objectives of this paper, three main themes were subject to focused analysis: mentees' experiences of OA, experience of peer mentorship support and factors influencing self‐management.

**Results:**

Seventeen mentees participated in an interview following completion of the peer support intervention. Themes emerging from focused analysis were the following: tackling the challenges of living with OA pre‐ and post‐intervention; and the interplay of the peer mentorship intervention and self‐management. Key elements of the latter theme are enabling factors provided by peer mentorship, and mentees' readiness to self‐manage.

**Conclusion:**

To effectively support OA self‐management, peer mentorship interventions should include core educational components and focus on strategies that enhance key enablers of self‐management. Paying attention to the mentor–mentee relationship and timing of intervention engagement can maximise opportunities for older people to adjust and transition from supported to independent self‐management.

## 1 INTRODUCTION

Osteoarthritis (OA) is a long‐term, age‐related musculoskeletal condition affecting around 8.75 million adults in the United Kingdom (Swain et al., 2020; Versus Arthritis, 2019). OA most commonly occurs in the hip and knee joints causing pain, stiffness and functional impairment. These symptoms impact significantly on daily living and quality‐of‐life particularly in older adults (Bay et al., 2020; Salaffi et al., 2005). With ageing populations, hip and knee OA present a high global burden of disease (Cross et al., 2014; Swain et al., 2020; Turkiewicz et al., 2014). Evidence supports the value of self‐management for long‐term conditions (Hughes et al., 2020; Silver, 2018; Turner et al., 2015) and the UK National Institute for Health and Care Excellence (NICE) guidance on the assessment and management of OA identifies self‐management as a feasible treatment option (NICE, 2014, 2015).

The principle of self‐management is that patients are enabled to become active partners in managing their health through appropriate provision of tools and techniques to address the day‐to‐day problems presented by their long‐term condition (Foster & Fenlon, 2011). Key components of effective self‐management programmes include problem solving, decision making and tailoring to individual needs (Lorig & Holman, 2003). A systematic review of chronic pain self‐management (Devan et al., 2018) highlighted feeling empowered, being supported and incorporating self‐management strategies in regular practice as being fundamental to success. Barriers included sustaining motivation to practice self‐management and symptom‐induced emotional stress. A systematic review of musculoskeletal pain self‐management interventions identified that programmes incorporating both psychological and exercise components were effective (Taylor et al., 2016) and that core elements of sustained self‐management include accommodation of coping strategies into everyday life, self‐acceptance and development of an identity other than ‘person with pain’.

Supported self‐management is an effective approach to coping with the challenges presented by OA. Successful self‐management requires that individuals become motivated to adopt helpful behaviours, have belief that their efforts are effective, be alert to symptom changes and confident that they can sustain self‐management beyond the supported term (Adams et al., 2017; Berry et al., 2021; Thorstensson et al., 2015). Inclusion of peers (people who share the same condition) enhances opportunities for individual relevance.

Peer mentorship is a type of self‐management support where an individual with lived experience (mentor) provides support and advice to another individual with the same condition (mentee). This type of ‘relational support’ appears critical to self‐management of chronic conditions and it ‘fuels all other types of support’ (Dwarswaard et al., 2016). Specific studies on peer mentorship interventions for chronic pain and inflammatory arthritis highlight the significance of personalised support and rewarding peer relationships to increase mentee engagement with self‐management (Matthias et al., 2015; Sandhu et al., 2013). Timing of interventions and individual readiness to self‐manage are important considerations for success (Blakemore et al., 2016; The Health Foundation, 2016).

We developed and trialled a peer mentorship intervention to support self‐management in older people with OA to assess: the feasibility of an OA peer mentorship intervention; and acceptability of the intervention to peer mentors and mentees. The feasibility study and qualitative evaluation of peer mentors' perspectives are reported elsewhere (Anderson et al., in press; Lavender et al., in press). This paper provides the qualitative exploration from the mentees' perspectives.

### 1.1 Aim

Our aim was to gain a clearer understanding of how the peer mentorship intervention can support OA self‐management. Specific objectives were to:

1. Identify mentees' perceived challenges of living with OA and explore how the peer mentorship intervention supported them in tackling these challenges
2. Explore mentees' experience of receiving the peer mentorship intervention and its impact on their OA self‐management

## 2 METHODS

This qualitative study used semi‐structured interviews with mentees to explore their views on study participation and the peer mentorship intervention. Consolidated criteria for reporting qualitative research guided analysis and reporting (Tong et al., 2007).

### 2.1 The peer mentorship intervention

Study participants receiving the intervention (mentees) were matched with a trained peer mentor who provided a programme of personalised structured support designed to aid OA self‐management. Peer mentors were older volunteers (>50 years) with a confirmed diagnosis of hip and/or knee OA. Their 2‐day training included practical, theoretical, and socio‐emotional topics around managing OA such as muscle strengthening, activity pacing, goal setting and connectedness; and incorporated mentoring skills (e.g., active listening and safe lone‐working). The training programme is reported elsewhere (Anderson et al., in press). Mentees were offered up to eight, weekly 1‐h support sessions in which they worked with their peer mentor to: learn about their condition and strategies for self‐management (e.g., embedding activity, pacing); identify individual support needs; and develop self‐management skills including identifying and setting achievable goals. All mentees were shown exercise techniques and encouraged to practice muscle strengthening with their peer mentor in the session and alone. Mentees were given handouts to refer to in‐between sessions. The intervention period ran between February and October 2019.

### 2.2 Recruitment

Mentees were invited by letter to take part in a semi‐structured interview following the end of their trial involvement. Twenty‐four study participants received the intervention, 21 agreed to be contacted about participating in an interview. This formed our convenience sample. Participant information packs were posted to these mentees and interviews were arranged. Two mentees did not respond, one declined to participate and one withdrew from the interview due to time constraints.

To explore the experiences of mentees at varying time points following completion of the intervention, interviews took place at either 8, 16 or 20 weeks following baseline.

### 2.3 Data collection

Semi‐structured interviews were conducted in person by an experienced qualitative researcher (EDR), previously unknown to mentees. Interviews took place at mentees' homes between August and November 2019 and followed a topic guide informed by previous literature and developed by the research team (Table 1). No subsequent changes were made to the topic guide although probes to encourage expansion of specific topics were introduced early on. Interviews focused on key elements of feasibility and acceptability of the overall study including perceptions of the peer mentorship intervention, experience of self‐management and recommendations for improvements.

**TABLE 1:** Interview topic guide with example questions

Participant interview topics
**Background:** experience of living with osteoarthritis, main challenges, pre‐intervention self‐management
How has having OA affected you in your day‐to‐day life? What changes, if any, have you made as a result of having OA?
**About the intervention:** experience of the intervention; most and least helpful aspects of the intervention; perceived impact on self‐management
Can you describe your overall experience of having peer mentor support? In which ways did you find peer mentor support most/least helpful? How has peer mentor support impacted on how you manage your OA now?
**About mentorship support:** experience of being matched with a peer mentor: perception of peer mentor guidance; possible improvements to peer mentor support
Can you tell me about your experience of being matched with your peer mentor? How might having a peer mentor be different from receiving an information booklet on managing OA?
**About study participation:** experience of participation in the overall Feasibility Trial
What was your understanding of the study and what it set out to explore? Overall, how satisfied were you with how the study was conducted? In your view is there anything that could be improved?

Abbreviation: OA, osteoarthritis.

Written consent was obtained prior to interviews. Mentees were assured of confidentiality and data anonymity. Interviews were digitally recorded with mentees' permission. Pseudo‐anonymised recordings were independently transcribed. Returned transcripts were checked for accuracy by EDR and interview feedback was shared within the team. Topics arising during interviews were discussed and points for further exploration were highlighted. Data saturation was assumed when no new topics or perspectives arose in subsequent interviews.

### 2.4 Data analysis

Interview transcripts were analysed using framework methods, allowing for team‐led transparency and flexibility (Gale et al., 2013; Ritchie et al., 2003). Familiarisation and systematic open coding of the data was undertaken individually by two experienced qualitative researchers (EDR and EL). Codes were generated independently through a process of constant comparison (Bradley et al., 2007). Points of divergence were discussed and agreed. This inductive process enabled identification of emergent categories forming the basis of the analytical frameworks used for charting. Coded data were entered into frameworks independently, but sense checked by both researchers. Triangulating researcher‐identified codes enhanced data credibility; validity was strengthened by repeated reflexive interrogation of the data by the researchers (Braun & Clarke, 2014; Korstjens & Moser, 2018; Spencer et al., 2013). Themes arising from the interview data were discussed with the wider research team and presented as preliminary findings at an early dissemination event attended by mentors and mentees. Feedback from this event prompted further interrogation of the data, refinement of themes and development of sub‐themes.

## 3 RESULTS

Seventeen mentees (11 women and 6 men) participated in semi‐structured interviews lasting on average 45.5 min (range 21–67 min). The mean age was 71.2 years (range 58–84). Notably, over half the sample had been living with OA for 5 or more years; most had OA in several joints; around two thirds lived alone, and the majority were retired (Table 2).

**TABLE 2:** Summary characteristics of interview participants

Characterisitic	*N* (%)
Gender	
Male	6 (35)
Female	11 (65)
Age (years)	
55–64	3 (18)
65–74	7 (41)
75–84	7 (41)
Marital status	
Married	7 (41)
Divorced	5 (29)
Widowed	4 (24)
Single	1 (6)
Employment status	
Employed	3 (18)
Retired	14 (82)
Area affected by OA	
Single hip	0 (0)
Single knee	2 (12)
Both hips	0 (0)
Both knees	6 (35)
Single hip and single knee	2 (12)
Single hip and both knees	4 (24)
Both hips and single knee	0 (0)
Both hips and both knees	3 (18)
Prior hip or knee replacement	
No	16 (94)
Yes	1 (6)
Duration of OA diagnosis	
<1 year	1 (6)
1–2 years	7 (41)
3–5 years	1 (6)
6–10 years	4 (24)
11–20 years	4 (24)

Abbreviation: OA, osteoarthritis.

Analysis of the complete interview data resulted in six themes relating to participation in the feasibility trial. To address the specific aims of this paper, the following three themes were subject to focused analysis:

1. Mentees' experience of living with OA
2. Experience of peer mentorship support
3. Factors influencing self‐management

Two important sub‐themes emerged which aide understanding of peer mentorship support in self‐management of OA: tackling the challenges of living with OA; and the interplay of the peer mentorship intervention and self‐management. The results reported here focus on these two sub‐themes. Mentees are referred to by pseudonyms to preserve anonymity. Duration since OA diagnosis has been included to add contextual meaning.

### 3.1 Tackling the challenges of living with OA

Mentees highlighted physical, emotional, practical, financial and social challenges resulting from OA. They predominantly reported that OA‐related pain and stiffness affected mobility. Walking was commonly problematic. Mentees reported challenges navigating steps, shopping, gardening and housework. Several had difficulties with dressing and disturbed sleep. Although challenges faced by mentees varied in severity, they were often pervasive and interconnected. One mentee was forced to leave her job due to debilitating OA symptoms, impacting on her socially and financially. Others feared financial consequences of being unable to work due to symptom deterioration. Typically, mentees were wary of aggravating OA by doing the ‘*wrong thing*’ and feared that everyday activity could have negative impacts long‐term.

*‘*And also fear around it thinking if I stand this long will it make it worse in the long run, so a lot of questions that I had that I didn't understand.’ (Dawn, OA 1–2 years)

Mentees highlighted the emotional impact of OA. They found persistent pain, stiffness and limited mobility distressing,

‘And it made me want to cry, you think God, is this it? Am I going to be so stiff, I can't even put my pants on, or my socks?’ (Rachel, OA 1–2 years)

Commonly, mentees were frustrated by a sense of restriction on their desire to remain physically and socially active.

‘Well, although I carry on with normal life, it isn't to the pace I would like it to be, and that's what I find most challenging.’ (Lorna, OA 11–20 years)

Several newly diagnosed mentees were dismayed by the sudden onset of symptoms and the invisible yet pervading nature of the condition.

‘So it's just your everyday life really that it affects so much and I think ‘cos people don't see it. I think that's one of the big things.’ (Ellen, OA 11–20 years)

Mentees who were unused to OA flare‐ups described them as baffling. Lessening of symptoms caused mentees to hope that their condition had improved, while sudden increase in pain or stiffness caused consternation.

‘I just wonder why sometimes I just take a few steps backwards. It should be going in the right direction.’ (Rachel, OA 1–2 years)

Those who were accustomed to variations in the severity of their symptoms approached activity pragmatically, for example, carrying a walking stick, and understood that better and worse times with OA followed a natural course.

‘…it has been in remission…but then, it will flare up and be very, very painful and debilitating’ (Lorna, OA 11–20 years)

Several mentees reported having at least one additional health condition including cancer, chronic back pain or heart disease. Coping with co‐morbidities may have resulted in the impact of OA being overshadowed for some, whereas for others the onset of OA was a significant event that prompted action.

‘No, no, I was that upset, I just thought, I'll try anything. If I can live healthier or at least still think I can get about, I have to do something’ (Dawn, OA 1–2 years)

#### 3.1.1 Tackling challenges pre‐intervention

Prior to taking part in the intervention, mentees reported five main approaches to tackling the challenges presented by OA:

1. Becoming less mobile to protect themselves from further pain
2. Maintaining general physical activity such as walking or cycling
3. Seeking advice from health professionals
4. Following physiotherapy prescribed exercises
5. Using regular medication to manage pain

Only one mentee reported modifying her diet to reduce flare‐ups and control weight. Several reported** **maintaining a pre‐established form of activity in order to manage OA. Others mentioned moderating their activity to avoid ‘overdoing’ it.

‘Yes, if I don't do any overstretching or any activity, it doesn't come at all.’ (Mark, OA 6–10 years)

A few mentees reported struggling to initiate changes related to managing their OA despite being advised to do so and believing it would help them.

‘Maybe if I did do some stuff that (health professional) told me to do. Like go to the gym, I think if I did go to the gym like we said we were going to go,…then I might feel a bit better in myself.’ (Karla, OA 1–2 years)

This mentee understood that she would likely benefit from becoming more physically active but lacked impetus to make a start. In contrast, another mentee persisted with a challenging exercise regime prompted by fear of symptom deterioration.

‘But I've got to a stage where I'm frightened to stop.’ (Rachel, OA 1–2 years)

Approximately two‐thirds of mentees had previously accessed physiotherapy, privately or through GP referral, although many suggested that they found the prescribed exercises too strenuous and difficult to sustain.

‘I'd done loads of going to physios and I think they give you exercises that are too difficult ’cause they're all about weights.’ (Ellen, OA 11–20 years)

Mentees understood that OA is not cured by exercise but some lacked belief that maintaining activity and practising exercises could improve their symptoms.

Around two‐thirds of mentees suggested that they regularly used over‐the‐counter medication to manage OA related pain and enable them to maintain daily activities.

‘I've learned the joys of paracetamol. It's a go‐to drug for me now.’ (Howard, OA 1–2 years)

Several mentees believed that a joint replacement was inevitable and the only effective long‐term solution to managing OA. This belief may have affected their willingness to engage in self‐management. One mentee reflected that, while surgery appeared the only possible long‐term option for her, she rejected it on the basis that her recovery would be unsuccessful as she lacked adequate resources.

‘My only cure is a hip replacement, something like that, but I don't have the facilities of [professional athlete] to sort of get over it.’ (Ruth, OA 6–10 years)

In summary, mentees' pre‐intervention self‐management ranged from no activity to challenging daily activity; taking regular and selective pain relief; modifying activity and diet in the hope that this would bring about relief. Mentees varied in relation to their long‐term and short‐term approach to self‐management and in their belief that OA symptoms could be modified by their efforts.

#### 3.1.2 Tackling challenges post intervention

Of the 17 mentees interviewed, twelve reported positive uptake of OA self‐management behaviours due to the peer mentorship intervention; two reported making limited changes to their behaviour and three mentees reported making no changes to their self‐management behaviour due to the intervention.

Mentees predominantly reported following recommendations to practise muscle strengthening exercises and increase their general physical activity for example, through walking, cycling or swimming. Those who were physically active pre‐intervention were encouraged to continue with their chosen activity. Mentees were helped to modify their ambitions to return to sport by trying a new activity (e.g., tai chi) or adopting a less strenuous alternative (e.g., walking football).

A significant change for many was introducing goals and working to achieve them. This helped mentees feel more in control of their OA symptoms, monitor their progress and sustain change for longer. Mentees who were able to develop regular exercise routines were encouraged by the positive results.

‘I get up in the morning and I do all the standing up ones before I even get a shower. During the day I do the sit‐down ones and then I go off for my walk, you see, I'm walking now, like I was…down to [town name], which is a mile, which I wasn't doing. So it's really been fantastic.’ (Ellen, OA 11–20 years)

Mentees gained understanding of the importance of planning activity and reported benefitting from pacing, a critical OA self‐management strategy.

‘I like to do walking, I like to get involved, I try and do the things that I used to do. But obviously I can't do them as much, but I do them slower, and pacing's important, yeah. I try and…I just think about it.’ (Dave, OA <1 year)

Four mentees reported making changes to their diet with the help of their peer mentor. Mentees demonstrated better understanding of their eating habits and how adopting healthier eating could positively impact on OA symptoms.

‘That was okay because she went through the exercises with me, and she went through some eating habits, so I changed my diet.’ (Mark, OA 6–10 years)

### 3.2 Understanding the interplay of the peer mentorship intervention and self‐management

The majority of mentees reported benefitting from mentorship support, although not all were motivated to self‐manage as a result of the intervention. Data on the challenges presented by OA, along with mentees' pre and post‐intervention responses, revealed that some mentees more readily engaged with the intervention, and some more readily made changes to self‐management behaviours. These groups were not mutually exclusive. Central to the interplay of the intervention and self‐management are two key concepts: enablers of self‐management and mentee readiness to self‐manage. In this context, enablers were the features of peer mentorship that enhanced uptake of self‐management behaviours, namely: improving understanding, building confidence and engendering motivation (Table 3).

**TABLE 3:** Peer mentorship enablers of self‐management behaviour

Enabling factor	Example of enabler	Illustrative quote
Enhancing understanding of OA and self‐management strategies	Making the intervention individually relevant Providing opportunities to clarify meaning Helping mentees understand their symptoms Helping mentees understand gain control	*‘She was great because she tailored it to me. She knew what I was like. After about three or four weeks we got…she knew exactly what I would do and I wouldn't do’* *‘And she explained, if I asked any questions she explained it and I learnt more about it to what I knew, I knew very little about osteoarthritis and I learnt quite a bit about it, what to do and what not to do and what will help and what won't help. And she were right, everything she told me, and I stuck to everything what she told me’* *‘We talk about good and bad days with pain, but how we can make it good, improve it a little bit if not good, improve it, that was what I learned from this research, that it's not the end you just sit and take tablet and then moan about your pain and grumble about your pain, but you can improve your pain’* *‘Because it gave me a new insight, that having the osteoarthritis, I don't have to just sit and accept it, and to say, well [mentee name], this is how it is, you're going to have pain, it's going to get worse, you're just going to sit. I could move away from that, and look at what I could be doing, where I could be going, what I could be involved in, to make it better for me. So, I think that's what I got from it’*
Improving confidence to undertake self‐management behaviours	Providing psychological reassurance Supporting safe and effective exercising Demonstrating authenticity and sharing experiences Helping mentees connect active self‐management and symptom improvement Helping mentees gain confidence to self‐manage	*‘I've got a positive attitude and I do things and I'm outgoing, it's lovely to have the reassurance of somebody else telling you these things, reiterating them for you. It's so reassuring is that, and I found her really helpful in that regard, and saying, yes, you're doing the right thing, and she did the exercises with me, which was lovely, and we both enjoyed doing them together’* *‘It's so much more beneficial having somebody who can tell you if you're doing the right thing or the wrong thing, and how far you should go’* *‘She's a person that you could sit and you could talk to and you could appreciate what she was saying, and I think she were genuine, she were very genuine. I think she'd had a pain problem before, and what she was sharing with me, she were genuine about it’* *‘I wouldn't have done as well, I don't think if I'd have not had a mentor, I'm sure, because she showed you the exercises and we gave one another tips on what to do to make situations easier…and I think that was really nice that she had the same experiences really’* *‘And then I think within—I don't know if it's one or two visits—I could straighten my knee. Now, I still can't with this one but it was just incredible…So that happened very quickly. And I think the rest has probably just built up. I feel stronger. I can tell that with [grandchild] because I can lift [grandchild] up now’* *‘I think it might give people confidence to go out more if they get more mobile, if you can get yourself more mobile ’cause it must be awful if you get so bad that you've got to stay in. That's my one fear, that I won't be able to go out’* *‘I'm a shy person, so one‐to‐one was very helpful, I was more confident, I was more relaxed to talk to’*
Engendering motivation to engage with self‐management behaviours	Setting and identifying progress towards goals Embedding exercise into regular routines Recognising the benefits of self‐management Feeling a sense of accountability	*‘Well, I knew they wouldn't be able to cure it. I were just hoping…I just wanted to find out more about it and how we could prevent it or make it easier. That was the goal for me’* *‘Well, the positive thinking, and knowing that I wanted to be active, and her pursuing that for me, or with me, and just motivation really, I found that she was good at. And I think that helped. Because she was asking what I wanted from it, and I said that I wanted to be as active as possible, and so that's what she tried to promote with me, and for me’* *‘I'm grateful that I were able to participate in that. I can see for myself that, if it's even the motivation, that is something, because I am motivated to do the exercise, and to do little walks, I am motivated, so even if it's just that, that is something’* *‘So, I think it just gave me that energy to get going again. Because you know somebody's coming the next week and wanting to know how you've got off it makes you do the exercises, but then I've realised the benefits that I'm getting down the line, so I think it just spurred me on again to get going’* *‘Everything I did, exercises I'd done and everything I…so that she'd give me a task every week. At the end of the thing she'd say, I want you to try and do this this week. I was accountable to her, so when she sat there, I didn't want to say to her I hadn't done it because she was very clever’*

Abbreviation: OA, osteoarthritis.

Working with their peer mentor, mentees were able to understand the information provided and clarify its relevance to them. They gained confidence to act on the information and were reassured that exercising correctly would not be harmful in the short‐term and would help in the longer‐term.

Through tailored support, mentees were helped to initiate self‐management behaviours, for example, practising muscle‐strengthening exercises, activity pacing, altering diet, engaging in outdoor activity. These initial behaviours were reinforced by peer mentor‐led motivators such as encouragement to set and work towards attainable goals and exercise daily; and mentee‐led motivators such as recognising changes to OA symptoms, feeling supported and gaining a sense of agency.

Supporting mentees to maintain self‐management behaviours longer‐term was fundamental to the success of the intervention. The role of the peer mentor was to enable mentees to initiate self‐management changes and to introduce strategies to foster mentees' motivation to sustain changes. However, the data suggests that translating initial changes into sustained change may be affected by mentee's readiness to self‐manage and the perceived value of the mentor–mentee relationship.

#### 3.2.1 The peer mentor–mentee relationship

The quality of the peer mentor–mentee relationship affected mentees' desire to engage with the intervention. Mentees enjoyed their peer mentorship sessions. They appreciated and often *‘looked forward’* to having time to talk and share concerns. Mentees enjoyed practising exercises with their mentor *‘We had fun actually’* and described developing a sense of connection through shared experience.

‘I enjoyed the time we had together, I could understand. Yeah, I appreciated her. Yes, I appreciated her very much.’ (Phyllis, OA 6–10 years)

Some expressed a sense of connection with their mentor beyond OA. Those who considered their mentor to be a positive role model were particularly invested in the intervention.

‘She had such a lovely attitude and disposition did [PM], whether I'd have felt like that with anybody else, I'm not entirely sure. But we did just gel.’ (Lorna, OA 11–20 years)

Typically, mentees wanted to emulate or please their mentor. Even mentees who maintained a more formal relationship with their mentor reported a sense of accountability developed through regular one‐to‐one contact. At least initially, this drove them to demonstrate their efforts to self‐manage.

#### 3.2.2 Readiness to self‐manage

Mentees' readiness to self‐manage was influenced by timing of their involvement in the study and the magnitude of their perceived OA challenge. For some, the invitation to participate was timely, coinciding with worsening OA or other health condition symptoms. These mentees were actively seeking change. Others appeared overwhelmed by their health concerns and welcomed support but were reluctant to initiate the changes recommended by their peer mentor during the intervention period. Some mentees were receptive to the intervention but were driven by curiosity rather than perceived need. These mentees tried out some self‐management strategies before reverting to their usual behaviour.

Mentees who were ready to self‐manage seemed to have accepted a longer‐term attitude towards OA. They reported that mentorship support gave them direction and impetus to renew activity goals.

‘It's made me more conscious to do the exercises, the physio exercises, and although I do walk and I am active, it's made me feel more strongly, that it is important to continue doing things like that.’ (Lorna, OA 11–20 years)

‘Well I think I've knocked on the head the idea that if my knees are bad that I should sit and do nothing’ (Howard, OA 1–2 years)

Mentees' perceived impact of OA on daily living, and fears for their future further shaped their attitude to self‐management. Those with mild symptoms and/or an established self‐management strategy were less eager to engage with the intervention than those living with severe symptoms and no self‐management plan.

‘No, I used to cry, honestly, in pain…I mean, it started in 40s, and then such a young age, what am I going to do next 10 or 15 years.’ (Shari, OA 6–10 years)

## 4 DISCUSSION

This qualitative study set out to explore acceptability and feasibility of a peer mentorship intervention from the mentees' perspective. The data provided insights into the challenges presented by OA, and mentees' self‐management response pre‐ and post‐intervention. In general, mentees enjoyed participation, gained practically and personally from interacting with their peer mentor and found content and format of support sessions workable. Focussed analysis of how mentees tackle challenges of OA alongside their intervention experience revealed that the impact of the peer mentorship intervention on self‐management is affected by the dynamic interplay of intervention and mentee‐related factors. Providing a robust peer mentorship intervention is only one component leading to effective self‐management. Other influential components include mentor–mentee relationships, readiness to self‐manage and key enablers. Understanding which can be moderated through the intervention and which are mediated by the environment is important to maximise opportunities for OA self‐management.

Consistent with other studies (Adams et al., 2017; Berry et al., 2021; Wilcox et al., 2006), we identified specific challenges of living with OA to include: limited knowledge of the condition, lack of clarity around symptom management, difficulty maintaining desired lifestyle and fears for the future. Intervention efficacy appeared to be shaped by the complex interaction of key elements: dominance of OA challenges; how far the intervention addressed individual support needs; perceived value of the peer mentor–mentee relationship and mentee's pre‐intervention approach to self‐management.

To better understand the variable impact of the intervention on mentees' self‐management, we explored which elements enabled an improvement in self‐management behaviours. We identified three important factors: enhancing understanding, improving confidence and engendering motivation. We noted that when these enablers were provided through peer mentorship support, there were positive changes to self‐management behaviour. However, this relationship is not linear and the impact of enablers on self‐management appeared to be enhanced or restricted by mentees' readiness to self‐manage and the mentor–mentee relationship.

When readiness to self‐manage was high and a good relationship was established, peer mentorship enablers had most impact on self‐management. Readiness to self‐manage is changeable and was greatest at initiation of the intervention, even for those with mild symptoms and existing self‐management strategies. The opportunity to convert initial uptake of self‐management behaviours into sustained changes is influenced by mentor–mentee relationship and evidence of symptom improvement (Simmonds et al., 2016). Our data suggest that the intervention had lower impact on mentees' self‐management for whom the enablers were less significant, for example, those who considered themselves to be knowledgeable about OA, who regularly engaged in physical activity and had strong existing social support. These mentees' readiness to implement new self‐management strategies was low.

A significant enabler of self‐management in this intervention was development of a supportive mentor–mentee connection. Individual in‐person delivery of the intervention was central to participants feeling cared for. Sharing experiences of OA and receiving personalised support helped mentors and mentees form a two‐way bond, which they found motivating. Mentees valued the advice they received and wanted to comply with their mentors' invitation to ‘*have a go’*. Even when the relationship remained more formal, it appeared to be mutually rewarding in that it was informative and/or enjoyable.

People with long‐term conditions have highlighted that working collaboratively with a healthcare provider, having consistent support and developing a positive attitude are effective enhancers of self‐management behaviours (Chaleshgar‐Kordasiabi et al., 2018; Nagelkerk et al., 2006). Participants in our study indicated that understanding OA and collaborating with their peer mentor better enabled them to self‐manage. Acting to control their symptoms and recognising the benefits of engaging in tailored self‐management improved confidence to continue with new strategies. Similarly, a qualitative study among patients with chronic pain/fatigue found the recognition of improved symptom management to be a facilitator in the continued implementation of activity pacing as a coping strategy (Antcliff et al., 2021). Those who found enjoyment in the mentor–mentee relationship were motivated to at least initiate self‐management behaviours. Motivation was further reinforced when the timing of the intervention coincided with higher readiness to self‐manage.

The findings of this study suggest that the success of an OA self‐management intervention is reliant on overcoming factors that hinder engagement. Studies have highlighted physical (e.g., exercise), psychological (e.g., attitude to health), educational (e.g., knowledge of condition), social (e.g., sharing experiences) and system (e.g., accessibility of support) factors as reasons not to engage with self‐management (Chen & Wang, 2007; Shakibazadeh et al., 2011; Wilcox et al., 2006). Our peer mentorship intervention was designed to address each of these factors whilst also focussing on factors that enable engagement with the intervention (Kosteli et al., 2017).

The one‐to‐one nature of the intervention enabled specific tailoring of mentorship support to mentees' needs. However, study time constraints meant that this support was not always offered to mentees at a time when they were most receptive to it. A real‐world setting would allow more flexibility around when the intervention is available. Using a readiness to change scale may be useful to identify mentees who are most likely to benefit from the intervention at a specific time. Conversely, using such a scale creates a risk of excluding some mentees with high support needs since it does not take into consideration the fluctuating nature of OA symptoms. Although the mentees in the study who were ready to self‐manage appeared to benefit most from the intervention, it is important to encourage self‐management in all individuals with OA.

### 4.1 Study strengths and limitations

A limitation of this study is that our sample lacked ethnic and socio‐economic diversity. However, the 17 participants showed variation in symptom range and severity, duration living with OA and response to OA challenges. Exploring self‐management changes with a larger, more diverse sample would improve generalisability.

We were unable to interview participants who dropped out of the intervention or declined an interview, so our findings are at risk of positive bias and should be considered with caution. To reduce bias, interviews were conducted by a researcher unknown to mentees and transcripts were double coded.

Participants were interviewed at a minimum of 2 weeks and maximum of 16** **weeks post‐intervention. This was insufficient time to establish whether self‐management changes instigated during the intervention were sustained, although mentees who demonstrated intrinsic motivation were potentially more likely to maintain self‐management behaviour longer‐term. A study incorporating longer follow‐up periods would be valuable.

A definitive Randomised Controlled Trial is required to provide evidence for real‐world practical and financial viability and address the limitations caused by study sample, time constraints and inclusion/exclusion criteria; and will allow us to better assess mentees' readiness to self‐manage OA. Definitive testing of the peer mentorship intervention would inform recommendations for wider implementation. We envisage that this intervention may require adoption by a patient organisation.

## 5 CONCLUSIONS

This qualitative study explored participants' attitudes towards and experiences of a peer mentorship intervention to support OA self‐management. The majority of mentees discovered value in the intervention and the mentor–mentee relationship, and they reported self‐management benefits. However, a minority reported *either* gaining information from the intervention *or* enjoying the social contact of mentor visits. Where only one of these elements was present, uptake of self‐management behaviours was less apparent.

Our findings suggest that effective peer mentorship interventions must include key components and must also focus on strategies that enhance key enablers of self‐management. We have demonstrated that the relational context and timing of mentorship support affects the intervention's effectiveness. We propose that paying attention to the enablers and moderators of productive peer mentorship relationships will create the best opportunity for early practical and psychological adjustment to self‐management, which in turn optimises the chances of older people transitioning from supported to independent self‐management.

## CONFLICT OF INTEREST

No potential conflict of interest was reported by the authors.

## ETHICAL APPROVAL

Ethical approval was granted from the Greater Manchester South Research Ethics Committee (Reference:17/NW/0238).

## AUTHOR CONTRIBUTIONS

All authors have contributed to the conception and design (Gretl A. McHugh, Philip G. Conaghan and Sarah R. Kingsbury) acquisition of data (Esther Dusabe‐Richards) or analysis and interpretation of data (Elizabeth C. Lavender and Esther Dusabe‐Richards). Authors Elizabeth C. Lavender, Anna M. Anderson, Deborah Antcliff and Gretl A. McHugh were substantially involved in the drafting of the manuscript and all authors critically reviewed it. All authors approved the final version, and take public responsibility and agree to be accountable for all aspects of the work.

## Data Availability

The data that support the findings of this study are available on request from the corresponding author. The data are not publicly available due to privacy or ethical restrictions.
